# Supplementary material for: The involvement of neuroimmune cells in adipose innervation
Source: Mol Med. 2020 Dec 9;26:126. doi: 10.1186/s10020-020-00254-3 (PMC7727151; doi:10.1186/s10020-020-00254-3)
Supplement: Supplementary file 1 — Additional file 1: Figure S1. Basal body and adipose of LysMCre±::BDNF−/− (KO) animals and gene expression of inguinal scWAT following cold exposure. Figure S2. Further phenotyping of LysMCre±::BDNF−/− (KO) animals. Figure S3. Assessment of BAT in LysMCre±::BDNF−/− (KO) animals. Figure S4. Adrβ3 gene expression in CINCs. [file 10020_2020_254_MOESM1_ESM.pptx]

## Slide 1
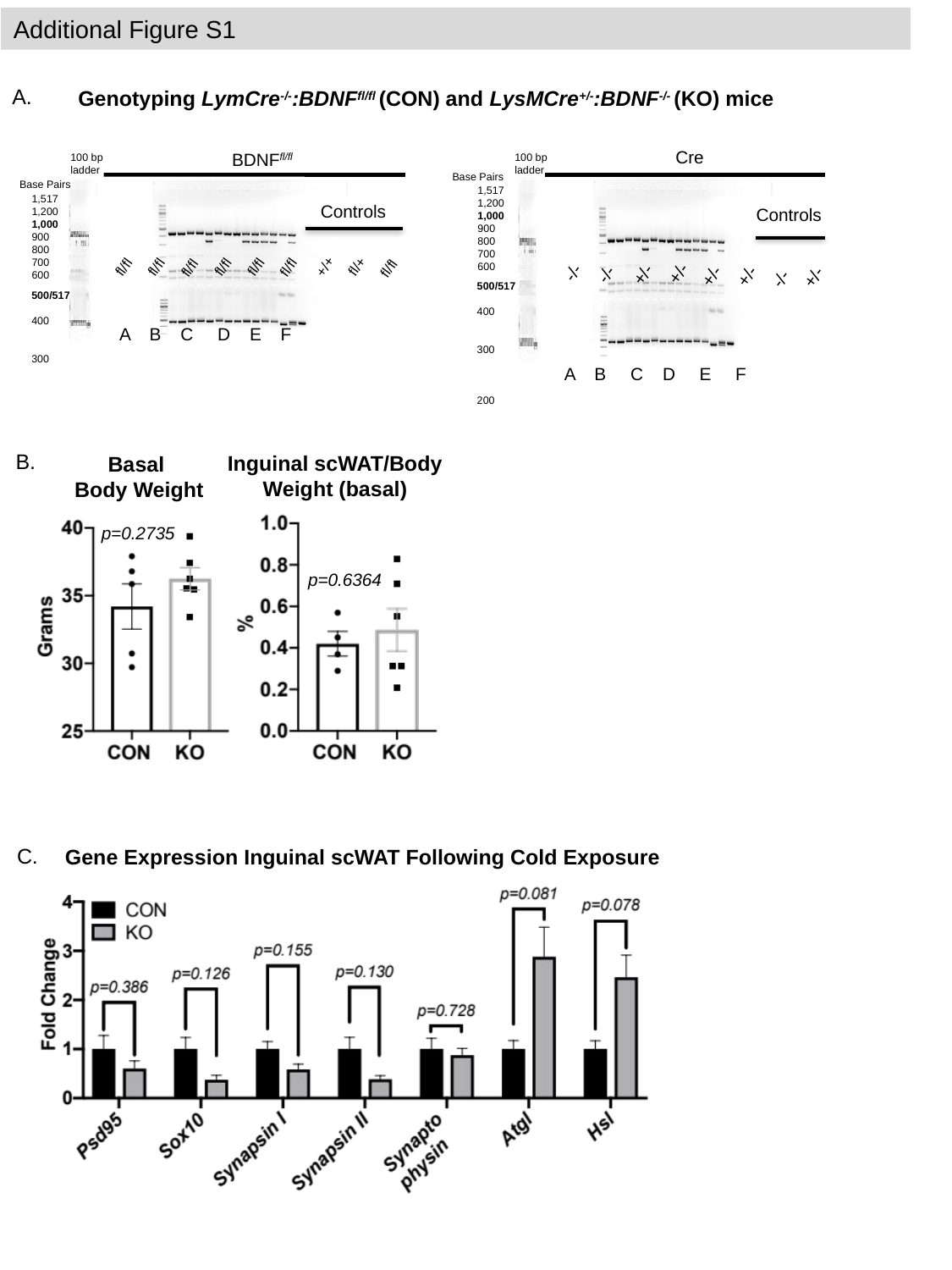

Additional Figure S1
A.
Genotyping LymCre-/-:BDNFfl/fl (CON) and LysMCre+/-:BDNF-/- (KO) mice
Cre
100 bp
ladder
Base Pairs
1,517
1,200
1,000
900
800
700
600
500/517
400
300
200
-/-
-/-
+/-
+/-
-/-
+/-
+/-
+/-
BDNFfl/fl
100 bp
ladder
Base Pairs
1,517
1,200
1,000
900
800
700
600
500/517
400
300
fl/fl
fl/+
fl/fl
fl/fl
fl/fl
fl/fl
+/+
fl/fl
fl/fl
Controls
Controls
A B C D E F
A B C D E F
B.
Inguinal scWAT/Body Weight (basal)
Basal
Body Weight
p=0.2735
p=0.6364
C.
Gene Expression Inguinal scWAT Following Cold Exposure

## Slide 2
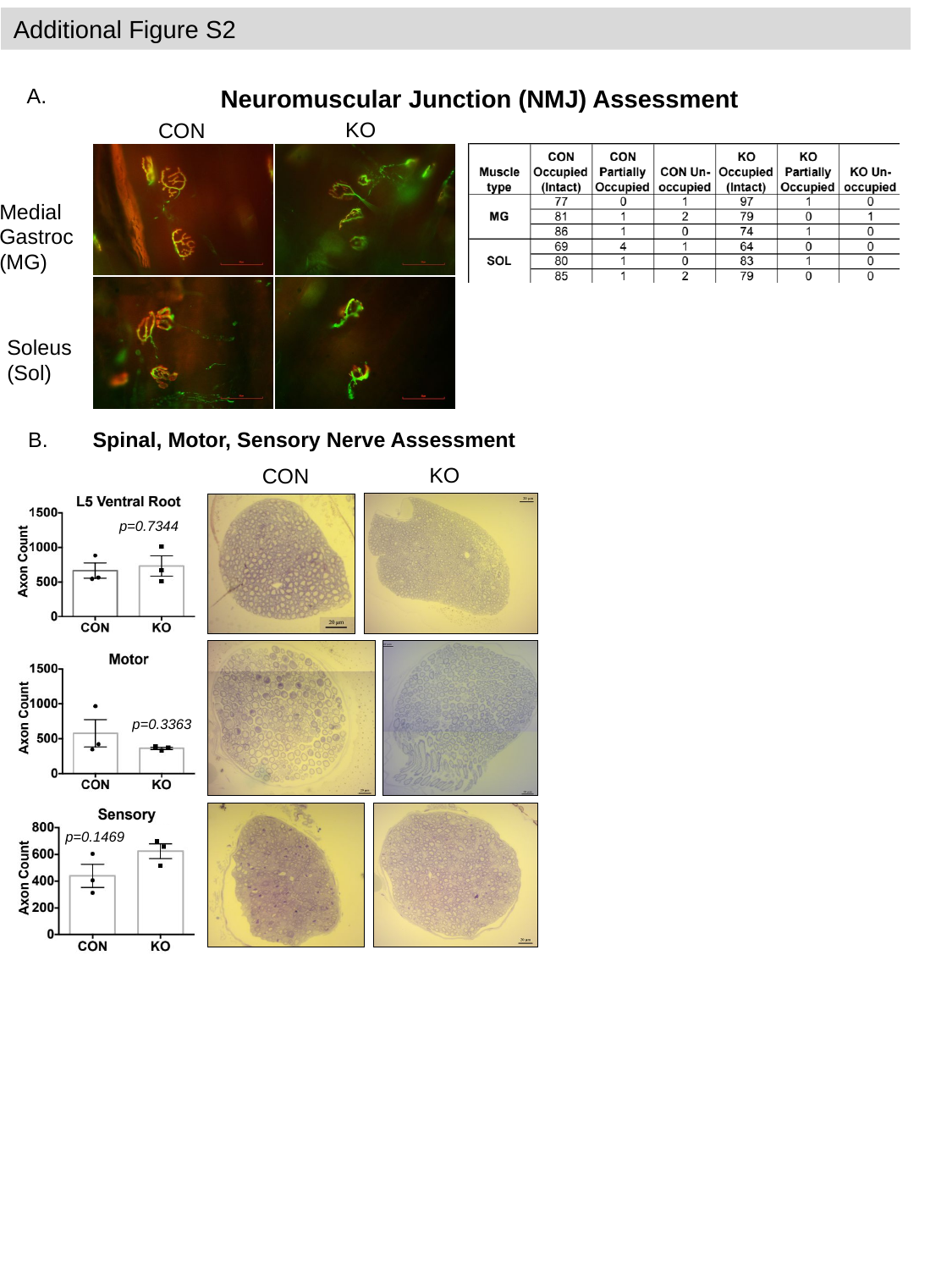

Additional Figure S2
A.
Neuromuscular Junction (NMJ) Assessment
KO
CON
Medial
Gastroc
(MG)
Soleus
(Sol)
Spinal, Motor, Sensory Nerve Assessment
KO
CON
B.
p=0.7344
p=0.3363
p=0.1469

## Slide 3
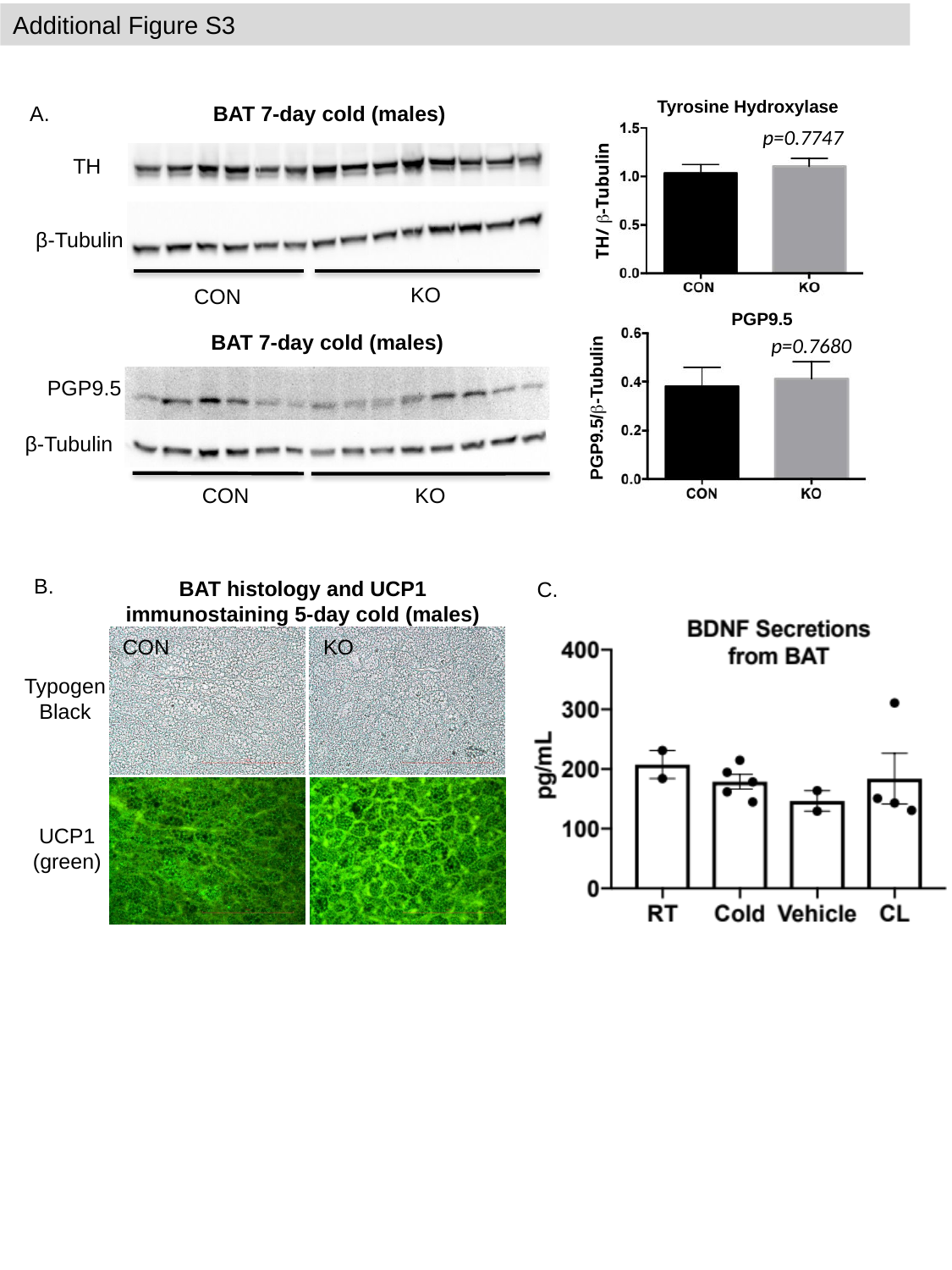

Additional Figure S3
Tyrosine Hydroxylase
A.
BAT 7-day cold (males)
TH
β-Tubulin
KO
CON
p=0.7747
TH/ β-Tubulin
PGP9.5
PGP9.5/β-Tubulin
BAT 7-day cold (males)
p=0.7680
PGP9.5
β-Tubulin
CON
KO
B.
BAT histology and UCP1 immunostaining 5-day cold (males)
CON
KO
Typogen Black
UCP1 (green)
C.

## Slide 4
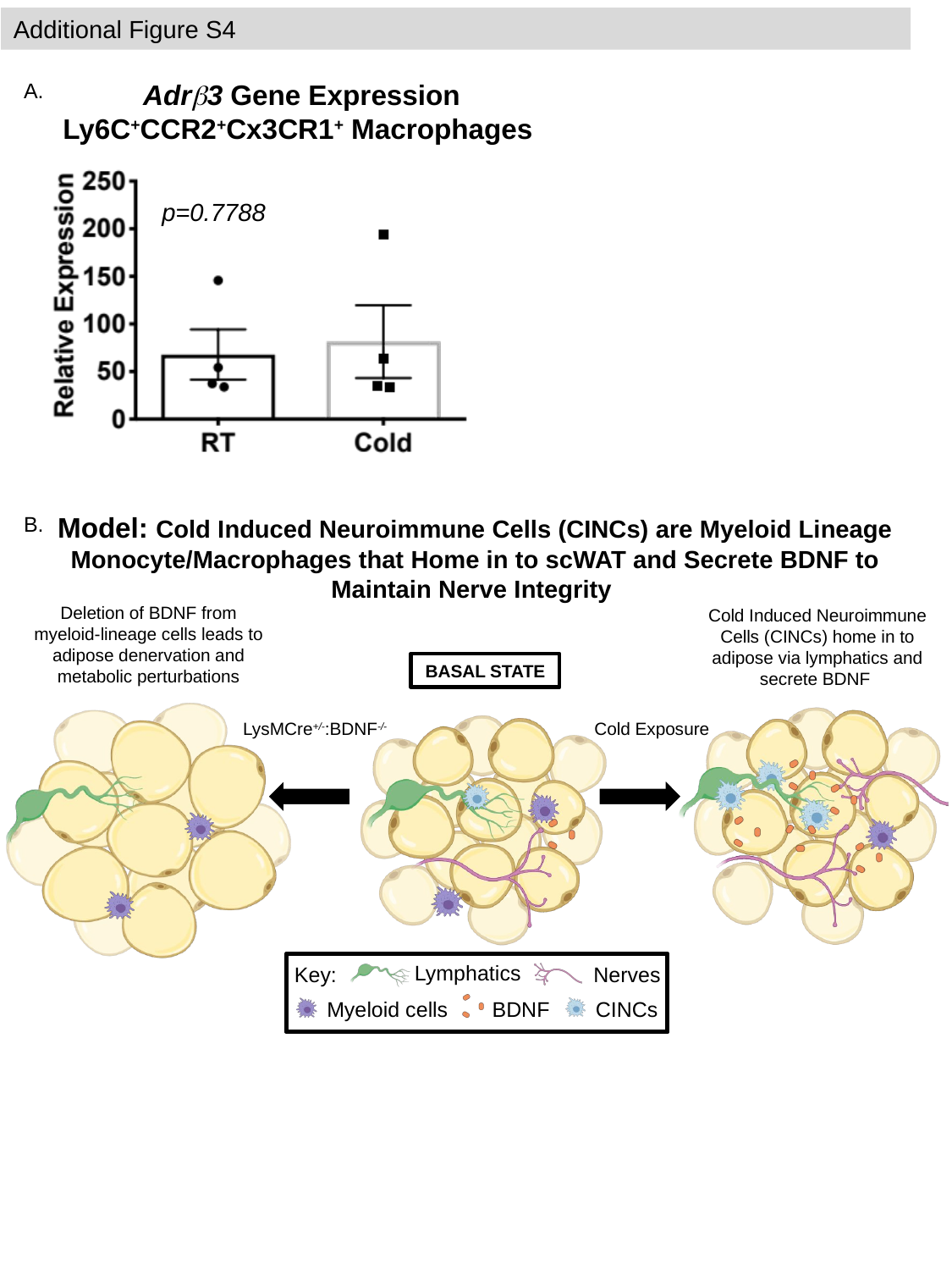

Additional Figure S4
A.
Adrβ3 Gene Expression
Ly6C+CCR2+Cx3CR1+ Macrophages
p=0.7788
Model: Cold Induced Neuroimmune Cells (CINCs) are Myeloid Lineage Monocyte/Macrophages that Home in to scWAT and Secrete BDNF to Maintain Nerve Integrity
B.
Deletion of BDNF from myeloid-lineage cells leads to adipose denervation and metabolic perturbations
Cold Induced Neuroimmune Cells (CINCs) home in to adipose via lymphatics and secrete BDNF
BASAL STATE
Cold Exposure
LysMCre+/-:BDNF-/-
Lymphatics
Nerves
BDNF
Myeloid cells
Key:
CINCs
